# Diffusion of digital breast tomosynthesis among women in primary care: associations with insurance type

**DOI:** 10.1002/cam4.1036

**Published:** 2017-04-04

**Authors:** Cheryl R. Clark, Tor D. Tosteson, Anna N. A. Tosteson, Tracy Onega, Julie E. Weiss, Kimberly A. Harris, Jennifer S. Haas

**Affiliations:** ^1^ Division of General Internal Medicine and Primary CareBrigham and Women's HospitalBostonMassachusetts; ^2^ Department of Biomedical Data ScienceGeisel School of Medicine at DartmouthLebanonNew Hampshire; ^3^ Norris Cotton Cancer CenterGeisel School of Medicine at DartmouthLebanonNew Hampshire; ^4^ The Dartmouth Institute for Health Policy and Clinical PracticeGeisel School of Medicine at DartmouthLebanonNew Hampshire; ^5^ Harvard Medical SchoolBostonMassachusetts; ^6^ Harvard T.H. Chan School of Public HealthBostonMassachusetts

**Keywords:** Access to care, cancer screening, digital breast tomosynthesis (DBT), digital mammography (DM), health disparities

## Abstract

Digital breast tomosynthesis (DBT) has shown potential to improve breast cancer screening and diagnosis compared to digital mammography (DM). The FDA approved DBT use in conjunction with conventional DM in 2011, but coverage was approved by CMS recently in 2015. Given changes in coverage policies, it is important to monitor diffusion of DBT by insurance type. This study examined DBT trends and estimated associations with insurance type. From June 2011 to September 2014, DBT use in 22 primary care centers in the Dartmouth ‐Brigham and Women's Hospital Population‐based Research Optimizing Screening through Personalized Regimens research center (PROSPR) was examined among women aged 40–89. A longitudinal repeated measures analysis estimated the proportion of DBT performed for screening or diagnostic indications over time and by insurance type. During the study period, 93,182 mammograms were performed on 48,234 women. Of these exams, 16,506 DBT tests were performed for *screening* (18.1%) and 2537 were performed for *diagnosis* (15.7%). Between 2011 and 2014, DBT utilization increased in all insurance groups. However, by the latest observed period, screening DBT was used more frequently under private insurance (43.4%) than Medicaid (36.2%), Medicare (37.8%), other (38.6%), or no insurance (32.9%; *P* < 0.0001). No sustained differences in use of DBT for diagnostic testing were seen by insurance type. DBT is increasingly used for breast cancer screening and diagnosis. Use of screening DBT may be associated with insurance type. Surveillance is required to ensure that disparities in breast cancer screening are minimized as DBT becomes more widely available.

## Introduction

Breast cancer is the second leading cause of mortality for women, and optimal strategies to detect early‐stage cancer—without increasing the risk for overdiagnosis—continue to be investigated. National Cancer Institute Surveillance, Epidemiology and End Results Program (SEER) data document higher risks for late‐stage cancer presentation and breast cancer mortality for women who are uninsured and who have Medicaid insurance, compared to commercially insured patients, underscoring the need to improve access to effective screening technologies in these vulnerable groups 1.

To this end, digital breast tomosynthesis (DBT) is potentially an effective technology to aid the detection and diagnosis of breast cancer. The DBT technique reconstructs multiple‐dimensional X‐ray images of thin breast tissue planes, and is thought to improve breast cancer visualization 2. In practice, early observational data suggest a benefit to adding DBT to conventional two‐dimensional digital mammography (DM), including improved cancer detection and reduced call‐back rates for false‐positive results 3, 4, 5. Breast cancer simulation models suggest adding DBT to DM may be cost‐effective in a clinical population of women with dense breast 6. Among women with heterogeneously and extremely dense breasts, the incremental cost of adding DBT imaging to screen for breast cancer has been estimated at $53,893 per quality‐adjusted life year, and after 12 rounds of screening, an estimated 0.5 deaths and 405 false‐positive findings per 1000 women might be avoided 6. The Food and Drug Administration (FDA) approved use of DBT in combination with DM for breast cancer screening for all women in 2011. However, at this writing, there are no clinical trial data to document the impact of DBT on mortality from breast cancer. The Centers for Medicare and Medicaid Services (CMS) recently approved reimbursement codes for DBT use, requiring no copayment for screening indications, although a copayment is required for diagnostic indications 7. Currently, performing DBT in combination with DM is considered experimental by some commercial insurers, and is not uniformly covered.

Clinical uncertainty over appropriate uses of DBT, and variation in insurance coverage could contribute to access disparities. In particular, it is not known whether insurance status contributes to early DBT utilization patterns, and whether disparities may exist for screening or diagnostic indications. Our current study uses data from 22 primary care centers to (1) describe the uptake of DBT imaging since FDA approval in 2011, and (2) investigate the role of insurance type as a potential driver of patterns of DBT utilization in practices with access to DBT.

## Methods

### Study cohort and setting

The study cohort included women from 22 primary care centers undergoing breast imaging at one of the nine affiliated radiology facilities that offered DBT in addition to conventional DM for screening or diagnostic purposes between June 2011 and September 2014, the most recent year for which data were available. Each facility was affiliated with one of the 22 primary care centers that participate in the Dartmouth (D) ‐ Brigham and Women's Hospital (BWH) Population‐based Research Optimizing Screening through Personalized Regimens (PROSPR) breast cancer research center. We analyzed data on mammograms (DBT and DM) performed among women aged 40–89 years old. The D‐BWH PROSPR research center studies primary care populations within the Dartmouth‐Hitchcock (DH) Health system in New Hampshire and the Brigham and Women's Hospital affiliated clinical network in Massachusetts.

### Data sources and definitions

To obtain information regarding mammogram type (DBT vs. DM) and indication for exam (screening vs. diagnostic), multiple data abstraction procedures were used including manual and natural language processing strategies to abstract data from radiology information systems (RIS), billing claim codes, and from the electronic health record (EHR). Descriptions of these data collection procedures have been published previously, and demonstrate successful identification of indication data for DBT mammography 8, 9. The insurance type (private/commercial insurance, Medicaid, Medicare, uninsured, or other payment) associated with each mammogram was obtained from the EHR.

We used EHR data and Census data to measure other covariates thought to influence DBT utilization: age at exam, breast density, race or ethnicity (non‐Hispanic white, non‐Hispanic black, non‐Hispanic Asian/Pacific Islander, Hispanic, and other/unknown race and ethnicity), the region of care where mammograms were performed, and neighborhood zip code median household income. Breast density was assigned according to the American College of Radiology Breast Imaging Reporting and Data System (BI‐RADS) criteria (almost entirely fatty, scattered fibroglandular density, heterogeneously dense, or extremely dense breasts) 10. Among women with an unknown breast density on their index mammogram, we retrieved the breast density from a prior mammogram. For women without a prior mammogram (5119 or 5.5% of the cohort), the index study mammogram was retained and analyzed as an “unknown” breast density. Five‐year estimates from the 2013 US Census American Community Survey 11 were used to classify neighborhood zip code median household income into quartiles based on the patient's residential address. A region of care indicator was included to denote that mammography was performed at a BWH imaging facility in Massachusetts or at a southern DH imaging facility in New Hampshire. A northern DH radiology facility was removed from this analysis because DBT was initially implemented for research purposes.

### Statistical analysis

In accordance with FDA‐approved use of tomosynthesis, all DBT screening and diagnostic exams were conducted with an accompanying DM exam. We reported the total number of mammograms performed for screening and diagnostic purposes, and the proportion of these mammograms performed as DBT during the study period, by patient characteristics. To describe the longitudinal patterns of DBT uptake by insurance category, we conducted a repeated measures analysis using generalized estimating equations (GEE), with the outcome being an indicator of the mammogram (conventional DM or DBT) being a DBT. This analysis accounted for women having multiple exams during the study period. We described DBT utilization at 6‐month intervals to examine diffusion of DBT use over time. We estimated associations between DBT use and insurance status within three periods of DBT uptake: early (June 2011 to June 2013), middle (July 2013 to June 2014), or late (July 2014 to September 2014). A logit link and binomial variance were assumed under an exchangeable working covariance structure. Separate analyses were done for screening and diagnostic indications. The PROC GENMOD procedure was used in SAS 9.4 (SAS Institute Inc. 2015. SAS^®^ 9.4, Cary, North Carolina, USA), with the LSMEANS option to produce predicted proportions of DBT exams by insurance and period.

## Results

During the study period, 93,182 mammograms were performed on 48,234 women (Appendix 1). Table 1 presents data on the proportion of DBT mammograms performed by study characteristics. The median age and interquartile range of the study cohort was 56 (49–65) years. Overall, 16,506 (17.7%) of mammograms were performed as DBT. The proportion of screening DBT was 18.1% of all screening mammograms (*N* = 76,994), and the proportion of diagnostic mammograms was 15.7% of all diagnostic mammograms (*N* = 16,188). Across all the years of study, DBT was used to perform mammography more frequently for women with private insurance coverage (19.5%) than for those with Medicaid coverage (17.7%), Medicare coverage (13.3%), and uninsured women (7.8%; Table 1). Overall, DBT was used more frequently for women with extremely dense breasts (19.2%), and women of non‐Hispanic Asian/Pacific Islander (20.6%) or non‐Hispanic white (19.6%) race, while a nonlinear pattern was observed for median neighborhood income based on residential zip codes (Table 1).

**Table 1:** Proportion of mammograms performed as DBT, by cohort characteristics and exam indication

	Proportion of mammograms performed as DBT^a^ *N* (%)		
Cohort characteristics	All mammograms *N* = 93,182	Screening mammograms *N* = 76,994	Diagnostic mammograms *N* = 16,188
All	16,506 (17.7)	13,969 (18.1)	2537 (15.7)
Age at Mammogram (years)			
40–49	6044 (23.7)	5079 (24.8)	965 (19.3)
50–74	9936 (16.2)	8483 (16.5)	1453 (14.6)
75 +	526 (8.6)	407 (8.3)	119 (9.8)
Race/ethnicity^b^			
NH white	12,667 (19.6)	11,039 (20.6)	1628 (14.7)
NH black	1153 (11.4)	867 (10.5)	286 (15.4)
NH Asian/Pacific Islander	614 (20.6)	531 (21.8)	83 (15.4)
Hispanic	1531 (12.7)	1081 (10.9)	450 (20.9)
Other/unknown	541 (15.5)	451 (15.5)	90 (15.7)
Insurance type^c^			
Private	12,070 (19.5)	10,459 (20.4)	1611 (15.2)
Medicaid	790 (17.7)	550 (15.5)	240 (26.8)
Medicare	2549 (13.3)	2079 (13.2)	470 (13.9)
Other	792 (20.1)	619 (19.0)	173 (25.3)
Uninsured	288 (7.8)	247 (8.1)	41 (6.3)
Breast density			
Fatty	619 (9.5)	504 (8.5)	115 (20.0)
Scattered fibroglandular	4520 (12.7)	3704 (12.4)	816 (14.2)
Heterogeneously dense	6906 (17.2)	5920 (18.5)	986 (12.3)
Extremely density	1122 (19.2)	939 (19.8)	183 (16.4)
Unknown	3339 (65.2)	2902 (65.6)	437 (62.8)
Neighborhood household income (zip code median)			
<$61,060	4374 (19.1)	3580 (18.8)	794 (20.5)
$61,060–$78,814	3847 (16.2)	3200 (16.3)	647 (15.7)
$78,815–$100,429	4440 (19.9)	3892 (21.0)	548 (14.5)
>$100,429	3642 (16.0)	3120 (16.8)	522 (12.7)
Unknown	203 (13.9)	177 (15.1)	26 (9.2)
Region of care			
BWH	8992 (11.0)	7016 (10.6)	1976 (13.1)
DH‐South	7514 (64.3)	6953 (65.4)	561 (53.7)

DM, digital mammography (conventional); DBT, digital breast tomosynthesis; BWH, Brigham and women's hospital; DH, Dartmouth‐Hitchcock.

a Proportions are calculated as the number of DBT exams divided by the total number of mammograms performed (DBT/(DBT + DM)). All mammograms (DBT and DM) for the cohort were performed where tomosynthesis capability was available during the study period between June 2011 and September 2014.

b The “Other” race category includes Native American and Alaskan Natives groups, which were of insufficient number to analyze separately.

c Of 93,182 mammograms, there were *N* = 29 mammograms with missing information for insurance status. “Other” insurance includes international payers, disability‐related insurance, and occupational accident‐related insurance and other payments.

**Figure 1:**
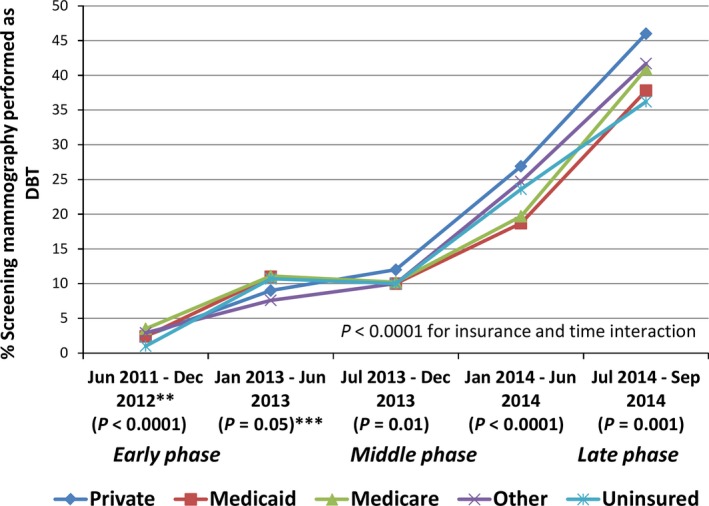
This figure shows time trends in utilization of screening DBT by insurance type. *Adjusted for age at mammogram, race, region of care (“BLINDED NAME”), breast density, and neighborhood household income. *^*^Due to small cell sizes the first time interval is 1.75 years. *^**^ *P*‐values for chi‐squared tests comparing insurance types at each time interval.

Figure 1 shows the longitudinal trend in DBT utilization for screening indication, by insurance type and adjusted for covariates. Across all insurance groups, use of screening DBT increased from 2.4% to 44.0% during the study period. The highest utilization increase was seen among exams covered by private insurance, from 2.5% in 2011 to 46.0% in 2014. Use of DBT for diagnostic purposes increased from 2.4% to 36.9% across all insurance groups; no sustained differences in use of diagnostic DBT were observed by insurance type (figure not shown.)

Table 2 shows early, middle, and late phase estimates of DBT utilization associated with insurance status for screening and diagnostic use, respectively. During the early phase of DBT uptake, a higher proportion of screening mammograms were performed as DBT among Medicaid (5.4%) and Medicare beneficiaries (3.5%) compared to those privately insured (2.8%) or uninsured (1.0%; *P* < 0.0001). However, by the late phase of DBT uptake, screening DBT was used more frequently under private insurance (43.4%) than Medicaid (36.2%), Medicare (37.8%), or the uninsured (32.9%; *P* = 0.001). Other factors associated with increased use of screening DBT were younger age, higher income level, and dense breasts.

**Table 2:** Proportion of DBT (95% CI) for insurance type by exam indication for each time period^a^^,^ b

	Screening indication	Diagnostic indication
	Proportion DBT (95% CI)	Proportion DBT (95% CI)
Insurance type		
Early phase: June 2011–June 2013	*P* < 0.0001	*P* < 0.0001
Private	2.8 (2.6–3.0)	3.9 (3.4–4.5)
Medicaid	5.4 (4.3–6.7)	5.0 (3.4–7.3)
Medicare	3.5 (2.9–4.2)	5.4 (4.2–6.9)
Other	3.9 (2.8–5.4)	7.0 (4.5–10.1)
Uninsured	1.0 (0.7–1.6)	1.5 (0.9–2.5)
Mid phase: July 2013–June 2014	*P* < 0.0001	*P* = 0.56
Private	16.8 (16.3–17.4)	21.9 (20.5–23.4)
Medicaid	14.1 (12.5–15.9)	25.2 (21.3–29.5)
Medicare	13.9 (12.9–14.9)	21.6 (19.1–24.4)
Other	15.7 (14.0–17.5)	23.3 (19.3–28.0)
Uninsured	12.5 (9.7–15.9)	25.7 (14.1–42.2)
Late phase: July 2014–Sep 2014	*P* = 0.001	*P* = 0.95
Private	43.4 (42.0–45.0)	36.0 (32.9–39.3)
Medicaid	36.2 (31.0–41.8)	35.9 (27.6–45.1)
Medicare	37.8 (35.0–40.6)	37.9 (32.5–43.6)
Other	38.6 (34.3–43.0)	36.0 (27.5–45.4)
Uninsured	32.9 (21.5–46.7)	29.9 (14.5–51.7)

DBT, Digital Breast Tomosynthesis.

a Models adjusted for age at mammogram, race, region of care (BWH, DH‐South), breast density, and neighborhood household income.

b *P*‐values for chi‐squared tests comparing insurance types at each time interval.

Similar insurance patterns of DBT uptake were found for diagnostic indication for the early phase. No insurance differences were found for diagnostic DBT in the middle (*P* = 0.56) and late phases (*P* = 0.99).

## Discussion

We found a rapid increase in the use of DBT in 22 primary care practices in Massachusetts and New Hampshire from 2011 to 2014. By late 2014, screening DBT use varied by insurance status during all periods, and in the late period, ranged from 32.9% among the uninsured to 43.4% among privately insured women. No sustained differences in diagnostic DBT use were seen by insurance status.

There are few data on utilization of DBT across the US. Data from a 2012 survey of physician radiologist members of the Society of Breast Imaging (SBI) estimated 30% of respondents used DBT for either screening or diagnostic purposes 12. The SBI data report that DBT use in this early phase of uptake was highest in academic medical practices and in the northeast US. Our results may reflect higher utilization compared to other regions of the country. Our findings of increasing disparities between uninsured, publically insured, and privately insured women may reflect uncertainty about methods for billing for this technology. Of interest from the 2012 survey, radiology respondents who used DBT reported that patients were required to sign a waiver accepting costs for DBT that were not covered by insurance, and that noncovered costs may have been charged to patients.

Our study has several limitations, including the geographic sampling frame, the inability to assess copayment charges, deductible levels for insurance plans, or other cost drivers of utilization that would give context to explain our results. Additionally, we note that the study period does not reflect the impact of newly assigned DBT billing codes from CMS, which may increase availability of DBT, and reduce uncovered costs for publically insured women. In our assessment of breast density as a correlate of DBT use, we were able to obtain breast density information on the vast majority of mammograms. However, we note that 4.1% of mammograms had breast density assigned based on a prior mammogram, which reflects a limitation of our data abstraction procedures. Breast density notification legislation is not likely to have played any role in the DBT utilization patterns we report since there was no legislation in effect within the study area throughout the study period, although MA enacted such a law that went into effect January 1, 2015.

Several strengths of our study include a longitudinal design, the ability to obtain direct measurement of mammography utilization from EMR and claims data, and a large sample with detailed measures of covariates that may influence estimates of the association between insurance type and DBT use.

We conclude that DBT is increasingly used to perform mammography in the settings we studied, consistent with earlier survey data suggesting increasing popularity of the technology. Additional data are required to recommend DBT as an approach for screening to improve health outcomes. However, we find that use of DBT for screening in primary care sites may be patterned by insurance status. Surveillance is required to ensure that insurance status differences in access to DBT do not contribute to disparities in breast cancer screening as DBT technology becomes more widely available.

## Conflict of Interest

No potential conflicts of interest were disclosed by the authors.

## Appendix 1

### Number of study participants (*N* = 48,234), and number of mammograms performed (*N* = 93,182), by women's characteristics at the time the mammogram was performed.

**Table:**

Women's characteristics	Total number of study participants *N* = 48,234 *N* (%)	Total number of mammograms (DM or DBT) *N* = 93,182 *N* (%)
Age at mammogram (years)		
40–49	14,709 (30.5)	25,518 (27.4)
50–74	30,591 (63.4)	61,529 (66.0)
75+	2934 (6.1)	6135 (6.6)
Race/ethnicity^a^		
White, non‐Hispanic	34,464 (71.5)	64,539 (69.3)
Black, non‐Hispanic	4847 (10.0)	10,091 (10.8)
Asian/Pacific Islander, non‐Hispanic	1601 (3.3)	2981 (3.2)
Hispanic	5622 (11.7)	12,084 (13.0)
Other/unknown	1700 (3.5)	3487 (3.7)
Insurance type^b^		
Private	33,180 (68.8)	61,935 (66.5)
Medicaid	1894 (3.9)	4453 (4.8)
Medicare	8819 (18.3)	19,110 (20.5)
Other	1634 (3.4)	3944 (4.2)
Uninsured	2685 (5.6)	3711 (4.0)
Breast density		
Fatty	2952 (6.1)	6499 (7.5)
Scattered fibroglandular	17,634 (36.5)	35,618 (40.8)
Heterogeneously dense	20,283 (42.1)	40,098 (45.0)
Extremely dense	2872 (6.0)	5848 (6.7)
Unknown	4493 (9.3)	5119 (5.5)
Neighborhood household income (zip code median)		
<$61,060	12,289 (25.5)	18,678 (24.6)
$61,060–$78,814	11,973 (24.8)	24,219 (25.5)
$78,815–$100,429	12,188 (25.3)	23,205 (23.9)
>$100,429	11,035 (22.9)	25,624 (24.4)
Unknown	749 (1.6)	1456 (1.6)
Region of care		
BWH	37,762 (78.3)	81,504 (87.5)
DH‐South	10,472 (21.7)	11,678 (12.5)

DM, digital mammography; DBT, digital breast tomosynthesis; DX, diagnostic; NH, non‐Hispanic; BWH, Brigham and women's hospital; DH, Dartmouth‐Hitchcock.

All mammograms (DBT and DM) for the cohort were performed where tomosynthesis capability was available during the study period between June 2011 and September 2014.

a The “Other” race category includes Native American and Alaskan Natives groups, which were of insufficient number to analyze separately.

b Missing (*N*): Insurance type (26 women, 29 mammograms). “Other” insurance includes international payers, disability‐related insurance, and occupational accident‐related insurance and other payments.
